# On the Measurement of Procrastination: Comparing Two Scales in Six European Countries

**DOI:** 10.3389/fpsyg.2016.01307

**Published:** 2016-08-31

**Authors:** Frode Svartdal, Gerit Pfuhl, Kent Nordby, Gioel Foschi, Katrin B. Klingsieck, Alexander Rozental, Per Carlbring, Sari Lindblom-Ylänne, Kaja Rębkowska

**Affiliations:** ^1^Department of Psychology, University of Tromsø – the Arctic University of Norway, TromsøNorway; ^2^Fakultät für Kulturwissenschaften, Fach Psychologie, Universität Paderborn, PaderbornGermany; ^3^Department of Psychology, Stockholm University, StockholmSweden; ^4^Centre for Research and Development of Higher Education, University of Helsinki, HelsinkiFinland; ^5^Department of Psychology, University of Warszawa, WarszawaPoland

**Keywords:** procrastination, scale, validation, measurement, cross-cultural

## Abstract

Procrastination is a common problem, but defining and measuring it has been subject to some debate. This paper summarizes results from students and employees (*N* = 2893) in Finland, Germany, Italy, Norway, Poland, and Sweden using the *Pure Procrastination Scale* (PPS) and the *Irrational Procrastination Scale* (IPS; Steel, 2010), both assumed to measure unidimensional and closely related constructs. Confirmatory factor analyses indicated inadequate configural fit for the suggested one-factor model for PPS; however, acceptable fit was observed for a three-factor model corresponding to the three different scales the PPS is based on. Testing measurement invariance over countries and students–employees revealed configural but not strong or strict invariance, indicating that both instruments are somewhat sensitive to cultural differences. We conclude that the PPS and IPS are valid measures of procrastination, and that the PPS may be particularly useful in assessing cultural differences in unnecessary delay.

## Introduction

When talking about goals, plans, intentions, and the intention-action-gap, it does not take long for the word procrastination to come up. “To voluntarily delay an intended course of action despite expecting to be worse off for the delay" (Steel, 2007, p. 66), is a common phenomenon that seems to be omnipresent in everyday life. It is widely studied in different disciplines of psychology. In fact, research on procrastination has somewhat exploded in the last decades, leading to a variety of approaches to defining and measuring it. This paper summarizes the results of a study that ran in parallel in six countries, in six different languages. The goal of the study was to compare the psychometric properties of two procrastination scales; examining factorial structure, internal consistency, item–test correlations, and convergent and discriminant validity, and to compare the scales across nations and between students and employees. Before going into the details of the study, we first characterize the phenomenon of procrastination and then present a short overview of the state of the art in measuring procrastination.

The different definitions of procrastination that circulate in the literature, all center on a few core aspects of the phenomenon (Klingsieck, 2013a). Of these, some pertain to the act being delayed: procrastination involves the delay of an overt or covert act that is necessary or of personal importance and where the start or completion was intended. The other aspects focus on the delay itself: it is voluntary and not imposed on oneself by external matters; it is unnecessary or irrational, meaning that it is carried out despite being aware of its potential negative consequences; while also being accompanied by subjective discomfort or even negative consequences.

The occurrence of procrastination depends on personal and situational factors. On the one hand, procrastination relates to personality traits such as conscientiousness and impulsiveness (Steel, 2007). It also occurs more often in the context of mastery-goal orientation (Howell and Watson, 2007), and less in self-determined activities (Senécal et al., 2003). On the other hand, certain task characteristics and contextual factors bear a greater potential for procrastination than others, for example, high task complexity (Ackerman and Gross, 2005), and the absence of clear deadlines (Schraw et al., 2007). Also, procrastination often entails manifest negative consequences concerning objective well-being, for example, health-related or academic achievement-related consequences (Tice and Baumeister, 1997) and subjective well-being (Deniz, 2006; Klingsieck, 2013b). Research indicates that approximately one-fifth of the adult population regard themselves as having great difficulties initiating or completing tasks and commitments (Harriott and Ferrari, 1996) while at least half of the student population perceive procrastination as a recurrent and severe problem in their everyday life (Day et al., 2000). However, albeit experienced as distressing, these numbers do not necessarily represent a clinical condition (Rozental and Carlbring, 2014), suggesting that only a small proportion is in need of psychological treatment. Moreover, no systematic comparisons between non-student and student samples have been made regarding their degree and character of procrastination, warranting further investigation to obtain more reliable estimates and possible qualitative differences.

### Measuring Procrastination

Although the concept of procrastination may seem quite straightforward, deriving valid methods for determining the degree of procrastination have proven to be quite complicated (Steel, 2010). Research has primarily relied on various self-report measures believed to entail a general trait or feature defined as procrastination, but often stemming from different theoretical frameworks (Rozental et al., 2014). An early attempt to devise an instrument was, for instance, the Decisional Procrastination Questionnaire (DPQ; Mann, 1982, unpublished; Mann et al., 1997), comprised of five items that capture the occurrence of putting off decisions, e.g., “I waste a lot of time on trivial matters before getting to the final decision” (item 9). Likewise, Schouwenburg (1995) introduced the Academic Procrastination State Inventory (APSI), having 23 items supposed to assess procrastination on a variety of study related activities, e.g., “Forgot to prepare things for studying” (item 11). Meanwhile, other instruments have also been developed, believed to evaluate the everyday tendency to procrastinate, such as the General Procrastination Scale (GPS; Lay, 1986), consisting of 20 items, e.g., “I generally delay before starting on work I have to do” (item 9), the Adult Inventory of Procrastination (AIP; McCown et al., 1989), encompassing 15 items, e.g., “I don’t get things done on time” (item 5), and the Aitken Procrastination Inventory (API; Aitken, 1982), containing 19 items, e.g., “Even when I know a job needs to be done, I never want to start it right away” (item 3). Given the moderate correlation between the GPS and AIP, however, it was assumed that they might involve different types of procrastination, arousal and avoidance (Ferrari, 1992), in line with the widespread notion of procrastination being caused by either a desire to seek thrills or experiencing performance anxiety (Klingsieck, 2013a). Steel (2010), however, performed a meta-analysis on prior studies of the instruments, as well as exploratory and confirmatory factor analysis (CFA) on data from a new sample responding to both scales, finding little evidence for this division. Instead, the results indicated that procrastination is probably more accurately conceived of as a unidimensional construct, general procrastination, accounting for a large degree of the variance, while some items were either unrelated to the definition of procrastination as a voluntary delay of an intended course of action, or loaded on different factors, in particular, those associated with being in a rush or being prompt.

Hence, Steel (2010) introduced a novel instrument, based on existing scales but consisting of only the 12 items that were directly related to general procrastination according to the factor analyses, the Pure Procrastination Scale (PPS). This scale was based on 12 items from three of the established procrastination scales discussed, the DPQ (PPS items 1–3), GPS (PPS items 4–8), and AIP (PPS items 9–12; Steel, 2010). The word “pure” reflects the fact that it has improved validity over previous instruments. The PPS is a self-report measure using a 5-point Likert scale (1–5), with higher scores indicating greater agreement. All items are consistent with procrastination, e.g., “In preparation for some deadlines, I often waste time by doing other things” (item 4). Furthermore, in line with the perception of procrastination as an irrational delay, Steel (2010) also introduced an additional instrument comprised of nine items named the Irrational Procrastination Scale (IPS), arguing that the PPS and the IPS should be closely related and thus be able to share validation efforts. The IPS is also a self-report measure using a 5-point Likert scale (1–5), with higher scores reflecting greater procrastination, e.g., “I put things off so long that my well-being or efficiency unnecessarily suffers” (item 1), and with the addition of three reversed items that are inconsistent with procrastination (items 2, 6, and 9). Moreover, although not directly assessing the same underlying construct, an instrument of impulsiveness and the tendency to give in to temptations was also proposed after being tested in relation to procrastination, the Susceptibility to Temptation Scale (STS; Steel, 2010). The STS is comprised of 11 items assessed on a 5-point Likert scale (1–5), with higher scores suggesting a greater tendency to become distracted by surrounding diversions, e.g., “When a temptation is right before me, the craving can be intense” (item 9).

Using a sample of 4169 individuals recruited via the Internet from English-speaking individuals across the globe, these instruments were shown to have high internal consistencies (PPS Cronbach’s α = 0.92, IPS α = 0.91, and STS α = 0.89), as well as convergent and divergent validity with other instruments of procrastination and one of well-being, the Satisfaction With Life Scale (SWLS; Diener et al., 1985), suggesting that the PPS and the IPS can be used in parallel. The STS exhibited a large average correlation with PPS and IPS, *r* = 0.69 (Steel, 2010), indicating that impulsiveness is strongly related to dilatory behavior, in accordance with prior findings.

Instruments such as the PPS and the IPS should in other words be more suitable in terms of determining the degree of procrastination given their increased convergent and divergent validity and improved correlations with other corresponding concepts (Klingsieck, 2013a). Also, the STS may be a useful instrument for examining impulsiveness and the tendency to give in to temptations, which, in turn, often results in procrastination. However, since their development and initial testing by Steel (2010), few attempts have been made to establish the properties of these instruments in other languages than English, as well as in relation to more diverse samples. Steel and Ferrari (2013) administered the IPS to a sample that was recruited via the Internet, but included only English-speaking individuals. Some or all of the instruments have been assessed in French (Rebetez et al., 2014), Swedish (Rozental et al., 2014), and Norwegian (Svartdal, 2015), but the results were restricted to treatment-seeking individuals, students, and adults, without systematic comparisons between samples, as well as obtaining somewhat different results between nations regarding factor structures of the scales. The French evaluation indicated that the PPS should be comprised of 11 instead of 12 items, suggesting a two-factor solution, with items 1–8 and items 9–11 loading on different constructs, “voluntary delay” vs. “observed delay” (Rebetez et al., 2014). The Swedish investigation, using a clinical sample (individuals being recruited for a clinical trial of internet-based cognitive behavior therapy for procrastination), obtained a two-factor solution for the PPS, one factor being more related to delaying decision making, not meeting deadlines, and missing appointments (PPS items 1–3 and 9–12), while the other was associated with starting late, lagging behind, and wasting time (items 4–8). In addition, the Swedish version of the PPS dropped the word “money” from item 12 and replaced it with the much broader notion “has cost me much.” Meanwhile, the IPS was found to encompass two factors, with the second factor involving only those items that were scored in reverse (items 2, 6, and 9), possibly reflecting a methodological artifact, while the STS only included one factor measuring susceptibility to temptation (Rozental et al., 2014). Regarding the Norwegian assessment, both the PPS and the IPS indicated one-factor solutions comparable to that of Steel (2010), albeit using a similar revision of item 12 for the PPS as the Swedish translation (Svartdal, 2015).

### The Current Study

To examine the utility of the PPS, the IPS, and the STS in a number of languages, as well as with regard to a more heterogeneous sample, the current study distributed the instruments to students and working individuals in six nations: Finland, Germany, Italy, Norway, Poland, and Sweden. The first objective was to conduct an evaluation of the different translations of the instruments using CFA based on initial findings by Steel (2010) as well as later results (Rebetez et al., 2014; Rozental et al., 2014; Svartdal, 2015). This evaluation addressed the factor structures of the instruments, and the PPS in particular. As noted, three different factor structures have been suggested for this scale. We argue that a fourth model should be examined. The PPS is based on items from three established procrastination scales, DPQ, GPS, and AIP. Examination of the DPQ and AIP items contained in the PPS indicate that they do not fit well to a definition of procrastination as irrational delay in implementation of intended action (Steel, 2007). Specifically, PPS items 1 and 3 address delay in decision (e.g., “I delay making decisions until it’s too late”; DPQ4), whereas PPS items 9–12 focus on deadlines and timeliness. For example, PPS item 9 “I find myself running out of time” (originally AIP10) may measure busyness and not procrastination *per se*, as noted by Steel (2010, p. 930) in discussing AIP items that were not included in the PPS. In effect, two of three DPQ items and all AIP items included in the PPS may not optimally measure the intended construct – “to voluntarily delay an intended course of action despite expecting to be worse off for the delay” (Steel, 2007, p. 66). Hence, evaluation of the PPS should address a fourth model for factor structure corresponding to three related but still different facets of delay: decisional delay, irrational delay of action, and delay in meeting deadlines and timeliness. The configural models of the various instruments were tested on the whole sample, and then for individual countries and the employee–student subgroups.

A second aim of the present study was to assess measurement equivalence over the participating countries. A few studies have compared procrastination scales between different English-speaking countries (e.g., Mann et al., 1998; Klassen et al., 2010), assuming but not assessing measurement equivalence. However, although construct (configural) equivalence as tested by CFA assures that the same construct is being measured by a given set of items, configural equivalence does not guarantee that scale means are comparable over nations or subgroups. For such comparison to be meaningful, weak (metric) as well as strong (scalar) equivalence must be present (Kankaraš and Moors, 2010; Brown, 2015). Metric equivalence requires that factor loadings for items in a scale are comparable across groups, and indicates that the construct has the same meaning over countries and subgroups; scalar equivalence requires that the scales are calibrated in the same way across nations and subgroups, i.e., that 1-unit increase in the latent construct has the same meaning over groups. Obviously, these requirements are threatened by cultural and subgroup differences in construct understanding, item biases, measurement errors, and method biases (Podsakoff et al., 2003; van Herk et al., 2004). Given satisfactory configural baseline models of the instruments, measurement equivalence can be evaluated by multigroup CFA (MG CFA) in increasingly restrictive steps, i.e., configural equivalence, structural (weak) equivalence, and measurement (strong) equivalence, and finally strict equivalence (Wu et al., 2007; Byrne, 2008; Brown, 2015; Kline, 2016).

Limited evidence is available to make sound predictions about equivalence over the participating countries. However, a number of observations indicate that procrastination instruments may be more vulnerable to cultural differences than is often assumed. For example, cross-cultural research on decisional procrastination (Mann et al., 1998) indicates substantial variations even between comparable cultures (i.e., USA, Australia, New Zealand). As decisional and behavioral procrastination are closely related (Mann, 2016), such differences may translate into cross-national differences in overall procrastination scores. Further, the six countries participating in this investigation, although all western, differ substantially in planning behaviors. For example, one study (Reinecke et al., 2013) found participants from Italy, Finland, and Sweden to plan ahead much less compared to those from Germany. Also, as procrastination is closely related to impulsiveness (Steel, 2007), cross-country variations in short-term discounting (Wang et al., 2016) speak for differences in impulsiveness and hence in procrastination over countries. In the Wang et al.’s (2016) study, 89% of German participants expressed a preference for delay in exchange for a bigger reward later, whereas the corresponding percentage for Italians was 44. Finally, gender differences (Steel and Ferrari, 2013) and differences between students and employees (Hicks and Storey, 2015) indicate that measurement equivalence over countries and subgroups may be uncertain. Overall, although the concept of procrastination is familiar in all countries participating in this study, thus satisfying a fundamental requirement of conceptual equivalence, we assume that the procrastination scales included in this study may be sensitive to cultural and subgroup differences.

## Materials and Methods

### Participants

A total of 2,893 students and working individuals from six countries; Finland, Germany, Italy, Norway, Poland, and Sweden participated. Participants were invited in lectures, by email invitations at institutes and institutions, and through social media. No incentives were offered for participation. All countries contributed with student samples > 200, Germany, Norway, and Sweden also contributed with employee samples >200; employee samples from the remaining countries are included in most analyses even if quite small (e.g., Poland and Italy). **Table 1** summarizes the number of respondents and subgroups for each country.

**Table 1:** Number of participants, students and employees.

	Total no. of participants	Students (females/males)	Students’ age, *M* (*SD*)	Employees (females/males)	Employees’ age, *M* (*SD*)
Finland	731	667 (572/95)	26.23 (6.93)	122 (100/22)	34.28 (11.88)
Germany	401	200 (153/47)	23.43 (4.18)	201 (148/53)	39.45 (10.40)
Italy	306	267 (211/56)	20.14 (2.52)	39 (31/8)	34.92 (8.21)
Norway	518	287 (191/96)	24.19 (5.95)	230 (170/60)	38.18 (10.67)
Poland	382	318 (257/61)	21.13 (2.35)	61 (58/3)	25.67 (4.88)
Sweden	555	251 (196/55)	26.73 (7.63)	302 (244/58)	39.45 (11.20)
Overall	2893	1990 (1580/410)	24.03 (6.13)	955 (751/204)	37.44 (11.13)

### Translating the PPS, IPS, and STS in Six Different Languages

Translations were made for the following languages; Finnish, German, Italian, and Polish, with the addition of the previously validated versions of PPS, IPS, and STS in Swedish and Norwegian. All of the translations were derived from the English instruments, originally developed by Steel (2010). For more information about the Swedish and Norwegian translations, please refer to Rozental et al. (2014) and Svartdal (2015). The translation of the other four language versions followed these four steps of translation and back translation: first, two persons (either the researchers themselves or English language and literature students) translated the English version into the target language. A few items had already been translated as part of other scales (e.g., the GPS, Lay, 1986). If these translations had already been published, these items were used instead of a new translation. Second, a third person fluent in both languages translated all items back into English. Third, this version was compared to the original version checking for meaning, content, and coherence. In this step, all three persons were involved. Fourth, the new language versions were discussed with the whole research team in order to check whether all items were interpreted in a similar manner by different persons.

### Instruments

The PPS consists of 12 items (Steel, 2010), all consistent with procrastination and rated on a 5-point Likert scale (1–5) with higher scores indicating greater agreement. Apart from its original validation, different translations of the PPS have since then obtained comparable results on samples of students, adults, as well as treatment-seeking individuals, α = 0.78–0.93 (Rebetez et al., 2014; Rozental et al., 2014; Svartdal, 2015). The IPS (Steel, 2010) features nine items, of which three are reversed and thus inconsistent with procrastination. Items are rated on a 5-point Likert scale, with higher scores (after reversal of the three procrastination-inconsistent items) indicating higher degree of procrastination. The Norwegian translation demonstrated good internal consistency, α = 0.85–0.93, and a high correlation with the PPS, *r* = 0.86 (Svartdal, 2015); the Swedish translation indicated somewhat lower values, α = 0.76, and *r* = 0.79 (Rozental et al., 2014). In terms of the STS, it differs somewhat from the other instruments as it is comprised of 11 items aimed to evaluate a single factor, susceptibility to temptations, or, impulsiveness, rather than procrastination *per se* (Steel, 2010). Items are rated on a Likert scale (1–5), with higher scores indicating higher impulsiveness. Impulsiveness as a personality trait has been found to be associated with dilatory behavior, *r* = 0.41 (Steel, 2007), suggesting that there is a close link between impulsiveness and procrastination. The Swedish translation of the STS achieved comparable results, α = 0.87, albeit with smaller correlations, *r* = 0.32–0.44, or, 0.39–0.53 when correcting for attenuation due to unreliability (Rozental et al., 2014). The Norwegian translation closely matched the original results (Steel, 2010), α = 0.87 and IPS-STS correlation *r* = 0.71.

Translated versions of the SWLS (Diener et al., 1985) were distributed with the instruments on procrastination. The SWLS consists of five items aimed to capture the subjective experience of global life satisfaction, e.g., “I am satisfied with my life” (item 3). The SWLS is a self-report measure using a 7-point Likert scale (1–7), with higher scores related to higher satisfaction with life. The SWLS was originally administered to samples of students and the elderly, with good internal consistency, α = 0.87, as well as a 2-month test–retest correlation coefficient of 0.82 (Diener et al., 1985). Subsequent investigations have obtained similar results, α = 0.79–0.89, with varying test–retest reliabilities depending on the time span, e.g., 0.51 for 5 years and 0.81 for 1 month (Pavot and Diener, 2008). The SWLS is available in a number of languages, including those used in the current study. In the present study, the SWLS yielded an overall internal consistency of α = 0.88, ranging from α = 0.84 (Italy) to 0.89 (Finland) in the individual countries.

### Ethics

The project of which the current study is part received ethical approval from the Regional Ethical Board in Tromsø, Norway (REK nord 2014/2313).

### Procedure

Data collection was performed over 2 months using an online survey system^1^. Participants were directed to a welcome web page, allowing them to select their native language introduction page. This page explained the purpose of the study, and that participation was anonymous and voluntary. The page also provided contact information to the country research site. Participants agreed to participate by actively pressing a start survey button. Once the survey was started, items on a given page had to be rated before proceeding to the next page. Mean completion time of the survey was 11 min.

### Statistical Analyses

Prior to analysis, all scales were examined for multivariate normality, and in particular, multivariate kurtosis which is known to be detrimental to parameter estimation in SEM (Byrne, 2008). Non-normality was apparent in each scale within each country according to the Mardia skewness and kurtosis tests. Hence, we used the Satorra–Bentler scaled chi-square statistic that adjusts for non-normality (Satorra and Bentler, 2001). The PPS, IPS, and STS were subjected to CFA, using the SEM module in STATA 14.1^2^. Initially, we evaluated configural fits to the suggested models in the complete sample as well as in the subsamples. Criteria to determine configural fit included the root mean square error of approximation (RMSEA), the Bentler comparative fit index (CFI), the goodness-of-fit index, and the standardized root mean square residual (SRMR; Byrne, 2001). In determining acceptable goodness of fit, we adopted the standard criteria of RMSEA < 0.08, CFI values in the 0.90–1.00 range, and SRMR < 0.08.

Next, to assess measurement invariance over countries and subgroups, we performed MG CFAs in R^3^ using the lavaan package (Rosseel, 2012; Hirschfeld and von Brachel, 2014). None of baseline models needed further adjustment although we noted a less good fit for the Finnish sample compared to the other countries. Measurement invariance was tested in four steps with the chi-square difference test between each successive step (Brown, 2015). In addition, the CFI differences between the models were examined. Cheung and Rensvold (2002) suggested that CFI differences should not exceed 0.01. The first step examined configural invariance across groups (equal form), the second step examined metric invariance (equal loadings), the third step examined scalar or strong equivalence (equal intercepts); finally, the fourth step examined strict invariance (equality of residual errors).

We also computed descriptive statistics for the IPS, PPS, STS, and SWLS for each participating country and for the two subgroups, employees and students. In addition, internal consistency (Cronbach’s α) of the respective scales and item-total correlations for each scale were computed, as well as correlations between scales to assess convergent and divergent validity.

## Results and Discussion

### Factor Structures of IPS, PPS, and STS

#### Pure Procrastination Scale

Three factor models have been suggested for the PPS: a one-factor model (Steel, 2010), a two-factor model with PPS items 1–8 (“voluntary delay”) and items 9–11 (“observed delay”), ignoring item 12 (Rebetez et al., 2014), and a two-factor model with items 4–8 (starting late, lagging behind, and wasting time on other things) and items 1–3 and 9–12 (focusing on delayed decision making, not meeting deadlines, and missing appointments; Rozental et al., 2014). Given the origin of the PPS items (see above), a three-factor solution corresponding to these different aspects was scrutinized as well.

As seen in **Table 2**, the first three models, and in particular, the one-factor model (Steel, 2010), did not demonstrate acceptable fit. On the other hand, the three-factor structure model for PPS in line with the origin of the items comprising it demonstrated an acceptable fit. Fit indices under this model for individual nations were acceptable for all nations except Finland. As the original one-factor model and the suggested three-factor models are nested, we performed chi-squared difference tests to test the null hypothesis of no difference between the models. The Satorra–Bentler χ^2^ change for the complete sample (1311.93, *df* = 2) was significant, *p* < 0.01, as were the changes within each country. CFI difference was 0.089. This indicates that a three-factor model of PPS is preferable to the original one-factor model. A corresponding analysis for the difference between the two-factor models and the three-factor model rendered similar results.

**Table 2:** PPS, CFA results for four suggested factor solutions.

	S_B scaled χ^2^	*df*	RMSEA_SB	CFI	SRMR
One-factor (Steel)	2060.11	54	0.113	0.866	0.059
Two-factor (Rebetez)	1083.65	43	0.092	0.925	0.049
Two-factor (Rozental)	1464.30	53	0.096	0.906	0.048
Three-factor model	748.18	51	0.069	0.955	0.037
Employees	186.33	51	0.053	0.974	0.028
Students	576.39	51	0.073	0.945	0.043
Male	153.86	51	0.058	0.965	0.037
Female	656.82	51	0.072	0.953	0.039
Sweden	131.52	51	0.053	0.965	0.033
Finland	385.05	51	0.095	0.929	0.060
Norway	203.12	51	0.076	0.946	0.047
Germany	135.34	51	0.064	0.959	0.042
Italy	106.23	51	0.060	0.958	0.048
Poland	96.26	51	0.048	0.979	0.031

*χ^2^ and RMSEA are Satorra–Bentler scaled.*

#### Irrational Procrastination Scale

The IPS was originally suggested to conform to a one-factor construct (Steel, 2010). Later, Rozental et al. (2014) proposed a two-factor solution, with items 2, 6, and 9 (all reversed in the scale) loading on a different factor compared to the other items. The results are shown in **Table 3**. Both models demonstrated acceptable fit, both on the complete sample and on the student–employee subsamples. Results from individual nations indicated acceptable fit for the one-factor model except for Finland and Sweden. Schmitt and Stults (1985) warned that reversed items may load on a separate factor due to inattentiveness, in the present case that some participants fail to detect the reversed meaning of items 2, 6, and 9. Rerunning the analyses excluding participants demonstrating inattentiveness did not alter the conclusions reported in **Table 3**.^4^

**Table 3:** IPS, CFA results for two suggested factor solution.

	S_B scaled χ^2^	*df*	RMSEA_SB	CFI	SRMR
One-factor (Steel)	523.71	27	0.080	0.958	0.032
Employees	182.89	27	0.078	0.964	0.032
Students	362.39	27	0.080	0.958	0.032
Females (2285)	493.84	27	0.087	0.953	0.034
Males (600)	78.61	27	0.056	0.976	0.028
Sweden	158.70	27	0.101	0.949	0.038
Finland	245.05	27	0.111	0.945	0.037
Norway	95.91	27	0.074	0.972	0.031
Germany	73.00	27	0.073	0.967	0.037
Italy	65.97	27	0.069	0.953	0.038
Poland	73.07	27	0.067	0.967	0.032
Two-factor (Rozental)	385.41	26	0.069	0.970	0.025
Sweden	125.34	26	0.083	0.961	0.031
Finland	206.40	26	0.098	0.955	0.033
Norway	71.85	26	0.058	0.982	0.024
Germany	57.55	26	0.055	0.975	0.031
Italy	54.36	26	0.062	0.965	0.034
Poland	62.62	26	0.061	0.977	0.029

*χ^2^ and RMSEA are Satorra–Bentler scaled.*

#### Susceptibility to Temptation Scale

STS is assumed to measure a single construct (Steel, 2010). The CFA indicated an acceptable fit with the complete sample, RMSEA_SB = 0.078, CFI_SB = 0.931, and SRMR = 0.037. Similar results were observed for the student and employee subsamples.

#### Satisfaction With Life Scale

For SWLS, the CFA indicated a good fit with for the complete sample, RMSEA_SB = 0.046, CFI_SB = 0.995, and SRMR = 0.013.

In summary, these data suggest a three-factor structure for the PPS, with PPS items 1–3 focusing on decisional delay, items 4–8 measuring “irrational delay” in behavior, and items 9–12 measuring delay in meeting deadlines and timeliness. IPS seems to measure a single construct, “irrational delay,” although it should be noted that the alternative model without reversed items demonstrated comparable fit indices. STS indicated support for the one-factor solution.

### Equivalence Over Countries and Subgroups

In assessing invariance over countries and subgroups, the three-factor model of PPS, and one-factor models of IPS and STS, were examined. For each instrument, invariance in four increasingly restricted steps were tested (i.e., configural, loadings, intercepts, and means).

#### Pure Procrastination Scale

For PPS, configural invariance across countries was observed, S_B χ^2^ = 1239.8, *df* = 306, RMSEA_SB = 0.080, CFI_SB = 0.951. Comparing countries pairwise yielded only one weak invariance (between Sweden and Italy); all other pairwise comparisons indicated configural invariance only. Notably, in all pairs the poorest fit was observed for items 9–12 (SE from 0.08 to 0.13). Next, we compared the employee to the student sample. Here we found weak invariance (RMSEA_SB = 0.071, CFI_SB = 0.955, difference to structural invariance deltaCFI = 0, deltaRMSEA = 0.003). Again, a closer look at the fits indicated the largest deviation among the two groups on items 9–12 (SE ranging from 0.053 to 0.057). Finally, we also compared females and males. Here we found minor loss of fits from configural, to weak, to strong, to strict invariance (structural RMSEA_SB = 0.076, CFI_SB = 0.953, strict RMSEA_SB = 0.071, CFI_SB = 0.951).

#### Irrational Procrastination Scale

The one factor model indicated structural invariance across the six countries, RMSEA_SB = 0.09, CFI_SB = 0.955. There was a weak invariance between Sweden and Poland, and between Germany and Italy, otherwise only structural invariance among the pairs of countries was observed. Items 3 and 9 had poorest fit across these pairwise comparisons. Next, we compared the employee to the student sample. Here, weak invariance was observed (RMSEA_SB = 0.08, CFI_SB = 0.957, difference to structural invariance deltaCFI = 0, deltaRMSEA = 0.005). Item 3 had the least fit (SE = 0.041). Finally, we also compared females and males. Here we found minor loss of fits from configural, to weak, to strong, and to strict invariance (maximal deltaCFI is 0.001), i.e., structural RMSEA_SB = 0.088, CFI_SB = 0.956, strict RMSEA_SB = 0.079, CFI_SB = 0.953.

#### Susceptibility to Temptation Scale

The one factor model yielded structural invariance across the six countries (RMSEA_SB = 0.091, CFI_SB = 0.933). There was weak invariance for the pair Sweden–Italy, else only structural invariance was found among the pairwise comparisons. The items with lowest fits varied and no pattern was found. Next, we compared the employee to the student sample. Here, we found weak invariance (RMSEA_SB = 0.083, CFI_SB = 0.929, difference to structural invariance deltaCFI = 0.001, deltaRMSEA = 0.004). Item 11 had the least fit (SE 0.041). Finally, we also compared females and males. Here we found only weak invariance, i.e., structural RMSEA_SB = 0.088, CFI_SB = 0.931, weak RMSEA_SB = 0.083, CFI_SB = 0.931.

Overall, these cross-cultural and group comparisons indicate that the procrastination instruments demonstrated the basic requirement for measurement invariance over countries and subgroups, i.e., configural invariance. For sex, strong invariance was observed both for PPS and IPS. As full scalar invariance is rarely observed in practice (e.g., Zercher et al., 2015), the lack of it need not imply that the scales cannot be used in different countries. However, an important implication is that comparisons and interpretations of mean differences across nations and groups cannot be done meaningfully. Note that poorest fit was observed on PPS items 9–12, indicating that these items are particularly sensitive to cultural variation.

### Relation between the Two Procrastination Scales

As the PPS and IPS attempt to measure the same construct using the same rating scale (Steel, 2010), procrastination scores from the two instruments should yield roughly equivalent scores. However, studies comparing these scales within language groups have consistently reported a high correlation between them but a considerable difference in mean scores (**Table 5**, Steel, 2010; Rozental et al., 2014; Svartdal, 2015) with lower PPS scores compared to IPS scores in the same subjects. As is seen in **Table 4**, PPS means in the individual countries were consistently lower compared to IPS means. Given the suggested three-factor solution for PPS, PPS item 4–8 means should correspond quite well to IPS. This is seen in **Table 4**. **Table 5** summarizes the Cronbach’s alphas and correlations between the scales and scale parts. Note that PPS items 4–8 correlate highly with PPS and IPS in all countries (*r* = 0.91–0.94, and 0.78–0.87, respectively). Hence, PPS items 4–8 achieve three purposes: they measure procrastination as well as the complete PPS, they correlate similarly with IPS, and they render a mean procrastination score quite comparable to IPS in all participating countries. In contrast, the correlations between these scales and PPS items 9–12 are considerably lower and vary substantially between countries.

**Table 4:** Means and standard deviations for PPS, PPS items 4–8, IPS, and STS for the participating countries.

	PPS	PPS 4–8	IPS	STS
Sweden	2.48 (0.73)	2.68 (0.90)	2.91 (0.80)	2.72 (0.77)
Finland	2.66 (0.74)	2.77 (0.88)	2.99 (0.79)	2.71 (0.72)
Norway	2.50 (0.78)	2.82 (0.98)	2.99 (0.79)	2.73 (0.74)
Germany	2.33 (0.77)	2.68 (0.99)	2.79 (0.78)	2.60 (0.79)
Italy	2.62 (0.67)	2.77 (0.86)	2.68 (0.65)	3.03 (0.59)
Poland	2.82 (0.81)	3.11 (0.99)	3.22 (0.81)	2.82 (0.72)

**Table 5:** Cronbach’s alphas and correlations (ranges over countries), PPS, IPS, PPS three-factor.

	Cronbach’s α	PPS	IPS	PPS 1–3	PPS 4–8
1. PPS	0.89–0.93				
2. IPS	0.85–0.93	0.79–0.87			
3. PPS 1–3	0.75–0.84	0.79–0.83	0.61–0.69		
4. PPS 4–8	0.88–0.93	0.91–0.94	0.79–0.87	0.63–0.71	
5. PPS 9–12	0.71–0.80	0.74–0.87	0.50–0.71	0.50–0.61	0.47–0.70

### Sex Differences

Overall, men demonstrated higher procrastination scores than women, but only marginally so. For example, the IPS mean scores were 2.91 vs. 3.04 for the two sexes, *F*(1,2883) = 11.703, *p* = 0.000, η^2^ = 0.004. This difference was stable across countries except for Finland and Poland, where no sex differences appeared. For the PPS subscales, an overall sex difference appeared only for PPS items 4–8, *F*(1,2883) = 6.31, *p* = 0.012, η^2^ = 0.002. This was seen over all countries except for Norway, where men had higher scores on all three subscales compared to women.

### Age

Procrastination correlates weakly and negatively with age (Steel and Ferrari, 2013), and this was observed in the present data as well. The correlation between age and IPS was generally negative and in the range *r* = -0.02 (Poland) to *r* = -0.23 (Norway). These correlations were lower or absent in students, *r* = 0.00 (IPS), higher in the employment group, *r* = -0.17 and *r* = -0.18, reflecting a restriction of range in the student group.

### Single vs. in a Relationship

Single individuals tended to procrastinate more compared to those married/in a relationship as measured both by the PPS and the IPS. This difference was stable in all countries except Finland, where no difference was observed, *F*(1,2874) = 1.756.

### Education

Except for Norway and Poland, participants with college/university education tended to demonstrate higher procrastination scores compared to participants with high school education.

### Procrastination, Impulsiveness, and Wellbeing

As expected, the STS correlated highly with the procrastination scales and moderately negatively with the SWLS, indicating convergent and divergent validity. Within each country, correlations between the STS and IPS ranged between *r* = 0.59 and 0.69, whereas the correlation between STS and SWLS ranged between *r* = -0.25 and -0.40.

## General Discussion

The purpose of the present study was twofold. First, using CFA, we evaluated the different translations of two well-established procrastination scales, the PPS and IPS (Steel, 2010) in order to assess their factor structures. Second, using MG CFA we compared the scales across countries and student vs. employee subgroups to assess measurement invariance. The main findings can be summarized as follows: (1) The PPS was found to conform to a three-factor solution corresponding to three aspects of delay, i.e., decisional, behavioral, and timeliness.^5^ (2) The middle part of the PPS scale (items 4–8) seems to address “irrational delay” in much the same way as does the IPS. (3) PPS items 9–12, all related to timeliness, seemed to be particularly sensitive to cultural and subgroup differences. (4) Overall, within-country comparisons confirmed previous findings that men procrastinate more than women, that students procrastinate more than employees do, and that single procrastinate more than individuals in a relationship, but even here cultural differences were apparent.

In effect, reliable and valid translations of the PPS and IPS can now be used in studies on procrastination, hopefully propelling research on cultural differences concerning procrastination that have been scarce in the literature. When implementing the PPS, however, it can be useful to bear in mind that items 9–12, all measuring timeliness, seem to be particularly sensitive to cultural differences. The six countries participating in this study differ markedly on Hofstede’s dimensions^6^ as well as in the dimensions measured in the World Value Survey (e.g., WVS wave 6). Notably, the six countries differ in individualism, which correlates with planning ahead (Reinecke et al., 2013). Finland, Germany, Norway, and Sweden are extensive users of scheduling tools whereas this is less prominent for Italy and Poland, and Italy was found to plan least ahead and Germany most (Reinecke et al., 2013). As such the individualism score of a country may influence the interpretation of the decisional delay items and also the timeliness items, which is another argument for separating the three parts of the PPS in cross-cultural comparisons.

Our findings indicate that there might exist a small sex difference, with men procrastinating more than women. Steel (2007) found similar results in his meta-analysis of 8756 participants. However, this difference was marginal, implying that this difference may not have any real-life implications. Also, the current study did not obtain the same results for the two samples that were recruited in Finland and Poland, suggesting that there might be other explanations for this difference than sex *per se*, e.g., cultural aspects or diverging social expectations for men and women. In addition, the effect of age on procrastination was observed in the current study, giving further support to the idea of decreased levels of procrastination with increased age (Steel and Ferrari, 2013). This is also in line with numbers showing that one-fifth of the adult population regard themselves as having difficulties of procrastination (Harriott and Ferrari, 1996), while at least half of the student population have recurrent problems completing their commitments. Why this is the case might be explained by a number of factors, and it has been argued that greater life experience results in less procrastination, as should the development of executive functions and changes in the perception of time, both of which are affected by age (Rozental and Carlbring, 2014).

With regard to the employee vs. student subgroups, the findings in the current study are similar to that of Steel and Ferrari (2013), indicating that being a student is associated with more procrastination. As noted, this may be related to age (Steel and Ferrari, 2013), but it could be important to consider contextual effects as well, that is, a work environment relies more on external control and predefined goals than an academic setting (Nguyen et al., 2013). Also, although the results were a bit inconclusive, a higher educational level was somewhat related to less procrastination, in accordance with Steel and Ferrari (2013), potentially reflecting a difference between the participants in terms of traits or features important for completing a higher academic degree, e.g., self-control (Steel, 2007). Similarly, being single as compared to in a relationship was also linked to more procrastination, as has been found in prior research (Steel and Ferrari, 2013). Again, the context might influence the degree of dilatory behavior, assuming that a partner makes sure that commitments are completed according to schedule and provides social support, which would limit the opportunity for delaying tasks and assignments. Furthermore, correlations between procrastination and different aspects related to well-being and mood have been found in several studies (Sirois et al., 2003; Sirois, 2007; Beutel et al., 2016), supporting the results in the current study where procrastination was linked to less satisfaction with life, as assessed by the SWLS (Diener et al., 1985). Given a definition of procrastination that emphasizes the negative consequences that delay may render, this association is not surprising. Although the relationship between procrastination and performance has been criticized for being inconsistent (Kim and Seo, 2015), it is never regarded as a particularly helpful behavior, often causing unnecessary stress, worry, and negative emotions (Steel, 2007).

As for the findings in the current study, several limitations and issues warranting further research need to be considered when reviewing the results. First, an obvious drawback pertains to the languages, and thus cultures, that were included. Although participants were recruited from different countries and settings, giving some credence to the generalizability of the results, cultural differences, particularly the timeliness aspect of procrastination, needs to be explored in more detail, using translations of the PPS and IPS in other languages. Second, as with all surveys and investigations implementing self-report measures, the risk of selection effects and biases has to be taken into account. Participants in the current study were invited in lectures, by email invitations at institutes and institutions, and through social media, making it possible to recruit individuals from a variety of different contexts. However, it is reasonable to suspect that those already experiencing difficulties of procrastination, or having a particular interest of this issue, participated to a greater extent, which may have influenced the results. In order to prevent some of these selection effects, the information given about the study did not contain any details regarding its aims, only instructing the participants to reflect upon their study or work habits. In terms of biases, social desirability might have occurred, making the participants respond in a way that is not perceived negatively by others, i.e., less problem with procrastination. Given the anonymity in the current study and that it did not include any type of feedback on the results, this risk should, however, be limited. Third, when measuring procrastination, more and more authors explicitly acknowledge the difference between procrastination and other forms of delay (e.g., Corkin et al., 2011; Grunschel et al., 2013; Klingsieck, 2013a; Krause and Freund, 2014). They agree that procrastination is an acratic, i.e., irrational, behavior pattern (cf. Andreou, 2007), while other forms of delay have a strategic nature. This acratic nature of procrastination is reflected by the conceptualization of the PPS and IPS. However, both scales fail to capture whether the delay has negative consequences for the participants. Furthermore, Steel et al. (2001) argue that self-report measures of procrastination may be influenced by a number of factors, most notably, unwillingness to provide an accurate account of one’s difficulties, as well as the impact of emotional states, such as, depression, anxiety, and low self-esteem, which could affect the subjective perception of not being able to complete tasks and assignments. In addition, Rozental and Carlbring (2014) discuss some of the drawbacks of relying solely on self-report measures of procrastination, including the issue of differentiating more severe and chronic procrastinators from trivial cases of putting something off, which is of particular importance in assessing the prevalence of procrastination in the general population. Embracing additional ways of determining the level of procrastination could therefore be of value, both in terms of more comprehensive self-report measures as well as other types of assessments with greater ecological validity.

## Author Contributions

All authors participated in data collection. FS and GP did the data analysis. KK, AR, and FS wrote the manuscript. All authors have read and suggested improvements in several iterations of the manuscript preparation.

## Conflict of Interest Statement

The authors declare that the research was conducted in the absence of any commercial or financial relationships that could be construed as a potential conflict of interest.

## Footnotes

1 www.Qualtrics.com

2 www.stata.com

3 www.r-project.org

4 To assess inattentiveness, we calculated a difference score between the mean of the procrastination-consistent IPS items and the reversed items. Including participants with <5 on this index (corresponding to a mean deviation of 1.67 per reversed item), CFAs still demonstrated acceptable fits for the one-factor model (S_B χ^2^ = 390.55, *df* = 27, RMSEA_SB = 0.070, CFI_SB = 0.97, SRMR = 0.025) as well as for the two-factor model (S_B χ^2^ = 378.66, *df* = 26, RMSEA_SB = 0.071, CFI_SB = 0.97, SRMR = 0.025). Similarly, a comparison of the two models with and without a common latent factor (Podsakoff et al., 2003, p. 168) indicated a negligible method effect.

5 The findings regarding the PPS three-factor structure are supported by independent data sets from published studies by Rebetez et al. (2014) and Rozental et al. (2014), who kindly provided us with data or analyses. In addition, Steel provided extensive data that support the three-factor PPS structure (Svartdal and Steel, in preparation).

6 www.geert-hofstede.com

## References

[B1] Ackerman D. S. Gross B. L. (2005). My instructor made me do it: task characteristics of procrastination. *J. Mark. Educ.* 27 5–13. 10.1177/0273475304273842

[B2] Aitken M. E. (1982). *A Personality Profile of the College Student Procrastinator.* Ph.D. dissertation, University of Pittsburgh Pittsburgh.

[B3] Andreou C. (2007). Understanding procrastination. *J. Theory Soc. Behav.* 37 183–193. 10.1111/J.1468-5914.2007.00331.X

[B4] Beutel M. E. Klein E. M. Aufenanger S. Brahler E. Dreier M. Muller K. W. (2016). Procrastination, distress and life satisfaction across the age range - a german representative community study. *PLoS ONE* 11:e0148054 10.1371/journal.pone.0148054 PMC4752450 26871572

[B5] Brown T. A. (2015). *Confirmatory Factor Analysis for Applied Research.* New York, NY: The Guilford Press.

[B6] Byrne B. M. (2001). Structural equation modeling with AMOS, EQS, and LISREL: comparative approaches to testing for the factorial validity of a measuring instrument. *Int. J. Test.* 1 55–86. 10.1207/S15327574IJT0101_4

[B7] Byrne B. M. (2008). Testing for multigroup equivalence of a measuring instrument: a walk through the process. *Psicothema* 20 872–882. 18940097

[B8] Cheung G. W. Rensvold R. B. (2002). Evaluating goodness-of-fit indexes for testing measure-ment invariance. *Struct. Equ. Modeling* 9 233–255. 10.1207/S15328007SEM0902_5

[B9] Corkin D. M. Shirley L. Y. Lindt S. F. (2011). Comparing active delay and procrastination from a self-regulated learning perspective. *Learn. Individ. Differ.* 21 602–606. 10.1016/j.lindif.2011.07.005

[B10] Day V. Mensink D. O’Sullivan M. (2000). Patterns of academic procrastination. *J. Coll. Read. Learn.* 30 120–134. 10.1080/10790195.2000.10850090

[B11] Deniz M. E. (2006). The relationships among coping with stress, life satisfaction, decision-making styles and decision self-esteem: an investigation with Turkish university students. *Soc. Behav. Pers.* 34 1161–1170. 10.2224/sbp.2006.34.9.1161

[B12] Diener E. Emmons R. A. Larsen R. J. Griffin S. (1985). The satisfaction with life scale. *J. Pers. Assess.* 49 71–75. 10.1207/s15327752jpa4901_13 16367493

[B13] Ferrari J. R. (1992). Psychometric evaluation of two procrastination inventories for adults: arousal and avoidance measures. *J. Psychopathol. Behav. Assess.* 14 97–110. 10.1007/BF00965170

[B14] Grunschel C. Patrzek J. Fries S. (2013). Exploring different types of academic delayers: a latent profile analysis. *Learn. Individ. Differ.* 23 225–233. 10.1016/j.lindif.2012.09.014

[B15] Harriott J. Ferrari J. R. (1996). Prevalence of procrastination among samples of adults. *Psychol. Rep.* 78 611–616. 10.2466/pr0.1996.78.2.611

[B16] Hicks R. Storey J. (2015). Can procrastination be effective? A study of white-collar employees and university students. *Int. J. Bus. Res.* 15:39 10.18374/IJBR-15-1.4

[B17] Hirschfeld G. von Brachel R. (2014). Multiple-Group confirmatory factor analysis in R–A tutorial in measurement invariance with continuous and ordinal indicators. *Prac. Assess. Res. Eval.* 19:2.

[B18] Howell A. J. Watson D. C. (2007). Procrastination: associations with achievement goal orientation and learning strategies. *Pers. Individ. Dif.* 43 167–178. 10.1016/j.paid.2006.11.017

[B19] Kankaraš M. Moors G. (2010). Researching measurement equivalence in cross-cultural studies. *Psihologija* 43 121–136. 10.2298/PSI1002121K

[B20] Kim K. R. Seo E. H. (2015). The relationship between procrastination and academic performance: a meta-analysis. *Pers. Individ. Dif.* 82 26–33. 10.1016/j.paid.2015.02.038

[B21] Klassen R. M. Ang R. P. Chong W. H. Krawchuk L. L. Huan V. S. Wong I. Y. (2010). Academic procrastination in two settings: motivation correlates, behavioral patterns, and negative impact of procrastination in Canada and Singapore. *Appl. Psychol.* 59 361–379. 10.1111/j.1464-0597.2009.00394.x

[B22] Kline R. B. (2016). *Principles and Practice of Structural Equation Modeling* 4th Edn New York, NY: The Guilford Press.

[B23] Klingsieck K. B. (2013a). Procrastination - When good things don’t come to those who wait. *Eur. Psychol.* 18 24–34. 10.1027/1016-9040/a000138

[B24] Klingsieck K. B. (2013b). Procrastination in different life-domains: is procrastination domain specific? *Curr. Psychol.* 32 175–185. 10.1007/s12144-013-9171-8

[B25] Krause K. Freund A. M. (2014). Delay or procrastination – A comparison of self-report and behavioral measures of procrastination and their impact on affective well-being. *Pers. Individ. Dif.* 63 75–80. 10.1016/j.paid.2014.01.050

[B26] Lay C. H. (1986). At last, my research article on procrastination. *J. Res. Pers.* 20 474–495. 10.1016/0092-6566(86)90127-3

[B27] Mann L. (2016). Procrastination revisited: a commentary. *Aust. Psychol.* 51 47–51. 10.1111/ap.12208

[B28] Mann L. Burnett P. Radford M. Ford S. (1997). The Melbourne decision making questionnaire: an instrument for measuring patterns for coping with decisional conflict. *J. Behav. Decis. Mak.* 10 1–19. 10.1002/(sici)1099-0771(199703)10:1<1::aid-bdm242>3.0.co;2-x

[B29] Mann L. Radford M. Burnett P. Ford S. Bond M. Leung K. (1998). Cross cultural differences in self-reported decision making style and confidence. *Int. J. Psychol.* 33 325–335. 10.1002/(SICI)1099-0771(199703)10:1<1

[B30] McCown W. Johnson J. Petzel T. (1989). Procrastination, a principal components analysis. *Pers. Individ. Dif.* 10 197–202. 10.1016/0191-8869(89)90204-3

[B31] Nguyen B. Steel P. Ferrari J. R. (2013). Procrastination’s impact in the workplace and the workplace’s impact on procrastination. *Int. J. Sel. Assess.* 21 388–399. 10.1111/ijsa.12048

[B32] Pavot W. Diener E. (2008). The satisfaction with life scale and the emerging construct of life satisfaction. *J. Posit. Psychol.* 3 137–152. 10.1080/17439760701756946

[B33] Podsakoff P. M. MacKenzie S. B. Lee J. Y. Podsakoff N. P. (2003). Common method biases in behavioral research: a critical review of the literature and recommended remedies. *J. Appl. Psychol.* 88 879 10.1037/0021-9010.88.5.879 14516251

[B34] Rebetez M. M. L. Rochat L. Gay P. Van der Linden M. (2014). Validation of a French version of the pure procrastination scale (PPS). *Compr. Psychiatry* 55 1442–1447. 10.1016/j.comppsych.2014.04.024 24920085

[B35] Reinecke K. Nguyen M. K. Bernstein A. Näf M. Gajos K. Z. (2013). “Doodle around the world: online scheduling behavior reflects cultural differences in time perception and group decision-making. *Paper Presented at the 2013 2nd ACM Conference on Computer Supported Cooperative Work, CSCW 2013* San Antonio, TX.

[B36] Rosseel Y. (2012). lavaan: an R package for structural equation modeling. *J. Stat. Softw.* 48 1–36. 10.3389/fpsyg.2014.01521

[B37] Rozental A. Carlbring P. (2014). Understanding and treating procrastination: a review of a common self-regulatory failure. *Psychology* 05 1488–1502. 10.4236/psych.2014.513160

[B38] Rozental A. Forsell E. Svensson A. Forsström D. Andersson G. Carlbring P. (2014). Psychometric evaluation of the Swedish version of the pure procrastination scale, the irrational procrastination scale, and the susceptibility to temptation scale in a clinical population. *BMC Psychol.* 2:54 10.1186/s40359-014-0054-z PMC4269972 25566392

[B39] Satorra A. Bentler P. M. (2001). A scaled difference chi-square test statistic for moment structure analysis. *Psychometrika* 66 507–514. 10.1007/BF02296192 PMC2905175 20640194

[B40] Schmitt N. Stults D. M. (1985). Factors defined by negatively keyed items: the result of careless respondents? *Appl. Psychol. Meas.* 9 367–373. 10.1177/014662168500900405

[B41] Schouwenburg H. C. (1995). “Academic procrastination: theoretical notions, measurement, and research,” in *Procrastination and Task Avoidance: Theory, Research, and Treatment* eds Ferrari J. R. Johnson J. L. McCown W. G. (New York, NY: Plenum Press) 71–96.

[B42] Schraw G. Wadkins T. Olafson L. (2007). Doing the things we do: a grounded theory of academic procrastination. *J. Educ. Psychol.* 99:12 10.1037/0022-0663.99.1.12

[B43] Senécal C. Julien E. Guay F. (2003). Role conflict and academic procrastination: a self-determination perspective. *Eur. J. Soc. Psychol.* 33 135–145. 10.1002/ejsp.144

[B44] Sirois F. (2007). “I’ll look after my health, later”: a replication and extension of the procrastination–health model with community-dwelling adults. *Pers. Individ. Dif.* 43 15–26. 10.1016/j.paid.2006.11.003

[B45] Sirois F. Melia-Gordon M. L. Pychyl T. A. (2003). “I’ll look after my health, later”: an investigation of procrastination and health. *Pers. Individ. Dif.* 35 1167–1184. 10.1016/s0191-8869(02)00326-4

[B46] Steel P. (2007). The nature of procrastination: a meta-analytic and theoretical review of quintessential self-regulatory failure. *Psychol. Bull.* 133 65–94. 10.1037/0033-2909.133.1.65 17201571

[B47] Steel P. (2010). Arousal, avoidant and decisional procrastinators: do they exist? *Pers. Individ. Dif.* 48 926–934. 10.1016/j.paid.2010.02.025

[B48] Steel P. Brothen T. Wambach C. (2001). Procrastination and personality, performance, and mood. *Pers. Individ. Dif.* 30 95–106. 10.1016/s0191-8869(00)00013-1

[B49] Steel P. Ferrari J. (2013). Sex, education and procrastination: an epidemiological study of procrastinators’ characteristics from a global sample. *Eur. J. Pers.* 27 51–58. 10.1002/per.1851

[B50] Svartdal F. (2015). Measuring procrastination: psychometric properties of the Norwegian versions of the irrational procrastination scale (IPS) and the pure procrastination scale (PPS). *Scand. J. Educ. Res.* 1–13. 10.1080/00313831.2015.1066439

[B51] Tice D. M. Baumeister R. F. (1997). Longitudinal study of procrastination, performance, stress, and health: the costs and benefits of dawdling. *Psychol. Sci.* 8 454–458. 10.1111/j.1467-9280.1997.tb00460.x

[B52] van Herk H. Poortinga Y. H. Verhallen T. M. (2004). Response styles in rating scales evidence of method bias in data from six EU countries. *J. Cross Cult. Psychol.* 35 346–360. 10.1177/0022022104264126

[B53] Wang M. Rieger M. C. Hens T. (2016). How time preferences differ: evidence from 53 countries. *J. Econ. Psychol.* 52 115–135. 10.1016/j.joep.2015.12.001

[B54] Wu A. D. Li Z. Zumbo B. D. (2007). Decoding the meaning of factorial invariance and updating the practice of multi-group confirmatory factor analysis: a demonstration with TIMSS data. *Prac. Assess. Res. Eval.* 12 1–26.

[B55] Zercher F. Schmidt P. Cieciuch J. Davidov E. (2015). The comparability of the universalism value over time and across countries in the European Social Survey: exact vs. approximate measurement invariance. *Front. Psychol.* 6:733 10.3389/fpsyg.2015.00733 PMC4455243 26089811

